# On the Application of a Diffusive Memristor Compact Model to Neuromorphic Circuits

**DOI:** 10.3390/ma12142260

**Published:** 2019-07-13

**Authors:** Agustín Cisternas Ferri, Alan Rapoport, Pablo I. Fierens, German A. Patterson, Enrique Miranda, Jordi Suñé

**Affiliations:** 1 Departamento de Física, FCEyN, UBA, Pabellón 1, Ciudad Universitaria, Buenos Aires 1428, Argentina; 2 Instituto Tecnológico de Buenos Aires, and National Scientific and Technical Research Council (CONICET), Buenos Aires 1437, Argentina; 3 Departament d‘Enginyeria Electrònica, Universitat Autònoma de Barcelona, 08193 Cerdanyola del Vallès, Spain

**Keywords:** memristor, compact model, emulator, neuromorphic, synapse, STDP, pavlov

## Abstract

Memristive devices have found application in both random access memory and neuromorphic circuits. In particular, it is known that their behavior resembles that of neuronal synapses. However, it is not simple to come by samples of memristors and adjusting their parameters to change their response requires a laborious fabrication process. Moreover, sample to sample variability makes experimentation with memristor-based synapses even harder. The usual alternatives are to either simulate or emulate the memristive systems under study. Both methodologies require the use of accurate modeling equations. In this paper, we present a diffusive compact model of memristive behavior that has already been experimentally validated. Furthermore, we implement an emulation architecture that enables us to freely explore the synapse-like characteristics of memristors. The main advantage of emulation over simulation is that the former allows us to work with real-world circuits. Our results can give some insight into the desirable characteristics of the memristors for neuromorphic applications.

## 1. Introduction

Memristive elements or resistive switches are two-terminal components that exhibit a hysteretic relation between voltage and current [1,2,3]. Because they are highly nonlinear and have the property of non-volatility, there is a great interest in their use in the design of new applications in neuromorphic circuits [4,5,6,7,8,9,10,11,12,13,14,15], programmable logic [16,17,18] and chaotic circuits [19,20,21], as well as in the development of new memory technologies [22,23,24,25]. Unfortunately, it is not simple to come by samples of memristors. Moreover, each time a researcher desires to adjust a parameter to change their response, she needs to go through a laborious fabrication and testing process. The usual alternatives are to either simulate [26,27,28,29,30] or emulate [31,32,33,34,35,36,37] the memristive systems under study. Emulation has the additional advantage that it allows to test the interaction of memristors with real circuit components [38]. For this reason, we present a simple emulator, based on widely-available and low-cost hardware that can work with various numerical models of memristors.

Since synapses can be understood as two-terminal elements with variable conductance, there has been an increasing interest on the application of memristors as synaptic junctions [4,5,6,7,8,13]. In particular, it has been proposed to modulate memristors conductance by applying specific signals, namely action potentials, with shapes and time characteristics that define the response of the neuromorphic circuit such as in the case of the Spike-Timing-Dependent Plasticity (STDP) process [6,8]. STDP relates the change in connection strength between two neurons as a function of the temporal distance between pre and postsynaptic stimuli [39,40,41]. It has been observed that the synaptic strength increases (decreases) when the presynaptic cell fires before (after) the postsynaptic neuron.

In the literature, we find some sophisticated protocols and complex circuits that allow for qualitatively mimicking the behavior of synapses [4]. However, it has been recently shown that the response of memristors with diffusive dynamics is already very similar to that of a synaptic junction [29,42]. Thus, it is interesting to study the application of this type of memristors in neuromorphic computing systems.

Memristors have also been used as part of more complex neuromorphic circuits. Particularly, in those that mimic the classical learning rule known as Pavlovian conditioning [43,44,45]. In this learning procedure, a specific stimulus that provokes a given response is paired with a neutral stimulus and, as a result of this pairing, the neutral stimulus can later evoke a response in the absence of the specific stimulus [46].

Since memristors with diffusive dynamics are well-suited to mimic the behavior of a synapse, in this work, we focus on the study of such type of memristors in simple neuromorphic circuits that present STDP behavior and Pavlovian conditioning, and study their performance as a function of the memristive device response time. To this aim, we consider a compact model of memristor that accurately describes the behavior of actual memristive systems [47,48,49]. This memristor model was implemented in an emulation architecture based on a microcontroller and a digital potentiometer. An exhaustive characterization of the emulator device and preliminary results of the STDP process were presented in Ref. [50].

### 1.1. Compact Model of Memristive Behavior

In this section, we review a compact model for memristors with diffusive dynamics that we have already introduced and have shown to represent accurately the experimentally measured behavior of actual devices [47,48,49].

Memristive devices are usually modeled by two equations. While one of the equations describes the I–V characteristic, the other governs the evolution of a state variable on which the I–V characteristic depends on. Many experimental reports show that, as multiple conductive channels in the insulator are created or destructed, metal-insulator-metal devices exhibit more than two conductive states. For this reason, we developed a model whose state variable tracks the fraction of active conductive channels [47,48,49]. Assuming that the channel creation probability follows a threshold distribution $f^{+}\left(v\right)$, the dependence of the creation of conductive channels $\Gamma^{+}$ on the applied voltage *v* can be calculated as

(1) $$\Gamma^{+}\left(v\right)=\int_{-\infty }^{+\infty }H\left(v-\xi \right)f^{+}\left(\xi \right)d\xi ,$$

where H(x) is the Heaviside function. On the other hand, the destruction of conductive channels $\Gamma^{-}$ can be obtained by considering the destruction threshold distribution $f^{-}\left(v\right)$. Both $\Gamma^{+}$ and $\Gamma^{-}$ are used to define a recursive formula for the discretized-time evolution of active conductive channels as

(2) $$\lambda \left(v\left(t\right)\right)=\min\left\{\Gamma^{-}\left(v\left(t\right)\right),\max\left[\lambda \left(v\left(t-h\right)\right),\Gamma^{+}\left(v\left(t\right)\right)\right]\right\}\;,$$

where *h* is the integration time step. The evolution of λ is highly sensitive to the creation and destruction distributions $f^{\pm }\left(v\right)$. For instance, skewed distributions may be suitable to describe devices where transitions take place abruptly upon reaching a given threshold potential while bell-shaped distributions may be used to describe those devices with gentle transitions [51]. For the sake of simplicity, we consider the latter approach with $f^{\pm }\left(v\right)$ following logistic distributions. Thus, the $\Gamma^{\pm }$ functions are given by sigmoid functions

(3) $$\Gamma^{\pm }\left(v\right)=\frac{1}{1+e^{-\alpha_{\pm }\left(v\mp \delta_{\pm }\right)}}.$$

**Figure 1:**
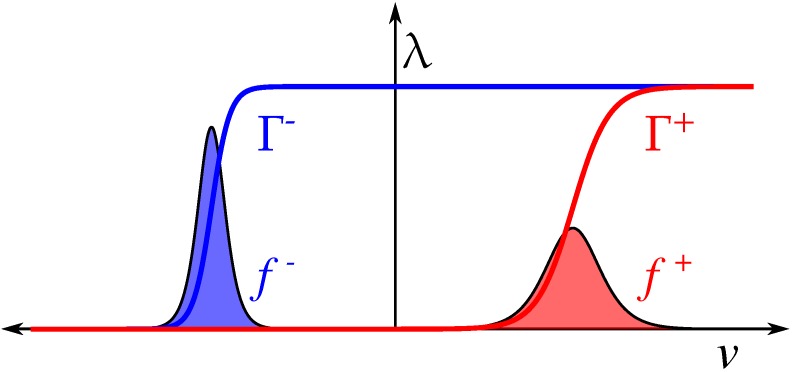
Threshold distributions $f^{\pm }$ are bell-shaped. The number of active channels λ is a function of the applied potential *v* and evolves within the region delimited by $\Gamma^{+}$ and $\Gamma^{-}$.

Parameters $\delta_{\pm }$ and $\alpha_{\pm }$ are positive constants that account for the positive and negative threshold potentials and transition rates, respectively. Figure 1 depicts voltage distributions $f^{\pm }$ and the corresponding $\Gamma^{\pm }$ functions. Equation (2) fixes the evolution of active channels λ between the region delimited by $\Gamma^{\pm }$. Conductive channels are neither aggregated nor dissolved instantaneously. Moreover, the response time depends on the magnitude of the driving signal. Experiments have shown that switching time and voltage are related by an exponential function. In order to account this phenomenon, the time evolution of active channels w(t) is described by the differential equation

(4) $$\tau_{0}\exp\left(-\frac{\left|v(t)\right|}{v_{0}}\right)\frac{d}{dt}w\left(t\right)+w\left(t\right)=\lambda \left(v\left(t\right)\right),$$

where $\tau_{0}$ is a characteristic response time, associated with a diffusive process, and $v_{0}$ a positive constant that weights the input stimuli.

The model is completed by specifying a relationship between w(t) and the I–V characteristics of the device. In previous works, the I–V characteristic equation was a nonlinear relationship that resembled that of two identical opposite-biased diodes [52,53]. As the main goal is to study the dynamics of switching effect, we simplified the I–V relation to that of a linear variable resistance described by

(5) $$R\left(t\right)=R_{\mathrm{on}}w\left(t\right)+R_{\mathrm{off}}\left(1-w(t)\right),$$

where $R_{\mathrm{on}}$ and $R_{\mathrm{off}}$ are the low and high-resistance levels of the memristor, respectively. This simplified relation alleviates the computation burden of simulation and emulation processes, without changing the essence of the model.

### 1.2. Emulation Architecture

**Figure 2:**
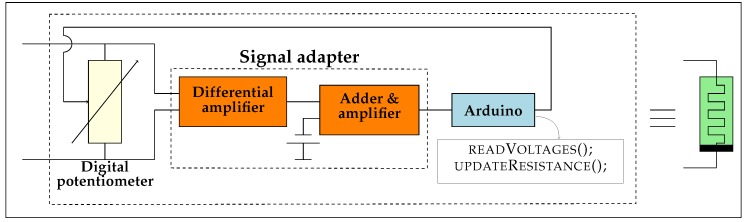
Schematic of the proposed emulator. An analog-to-digital converter (ADC) in the microcontroller measures the voltage on the digital potentiometer. The differential equations describing the memristor behavior are numerically integrated based on those measurements; then, the potentiometer resistance is changed accordingly. Signal conditioning is required to adapt the voltage to the microcontroller ADC input range.

Many emulation architectures have been proposed [31,32,33,34,35,36,37]. Following the work of Olumodeji and Gottardi [36], we base our design on an Arduino board and a digital potentiometer. A schematic of the emulator design is shown in Figure 2. The analog-to-digital converters (ADCs) in the Arduino are used to measure the current that flows through the potentiometer. The microcontroller integrates the differential equations that model the behavior of the memristor and changes the resistance of the potentiometer. A description of the emulator architecture is given in Section 3.1. In Section 2.1, we present results that validate the correctness of the implemented emulator.

### 1.3. Memristors for Neuromorphic Applications

There is a vast literature on the application of memristors for machine learning and computation (see, e.g., [54,55,56,57,58] and references therein). In particular, a review of the pertinent bibliography reveals a special interest on the use of memristors in neuromorphic circuits [4,5,6,7,8,9,10,11,12,13,14,15]. The focus of this paper is to study the possibility of using memristors as neuronal synapses and to characterize the role of the parameters that influence the dynamics of the memristive behavior.

A synapse is a biological structure that allows the communication of two neurons which is located, for example, in the junction of an axon with a dendrite of a different cell. The modulation of the synaptic strength plays a crucial role in learning and memory formation. By adjusting the weight of cells’ connections, the neural network can be reconfigurated. The connection strength between network elements is adapted through processes known as learning rules. One type of these processes is the one described by the Hebbian theory where it is postulated that the synaptic modulation is driven by correlations between pre and postsynaptic neuronal activity. Spike-Timing-Dependent Plasticity (STDP) process [39,40,41,59] is one common protocol to analyze the adaptation of synaptic strength. The initial strength of connection is quantified by measuring the response of the postsynaptic neuron to the application of a measurement pulse to the presynaptic cell. Then, a periodic sequence of presynaptic and postynaptic stimuli, separated by a time Δt, is applied. The effect of such stimuli signals is evaluated by measuring the postsynaptic response to a new test pulse in the presynaptic neuron. STDP describes the dependence of the change of synaptic strength, before and after the treatment, on Δt. In Section 2.2, we review results of a series of experiments [50] with emulated memristors that mimic the STDP process of real biological synaptic junctions.

Classical conditioning is another type of learning theory that relates preceding stimuli and behavioral reactions in animals. Let us assume that there is an unconditioned stimulus (US) that provokes an unconditioned response (UR). There is also a neutral stimulus (NS) that initially does not provoke any response. If the neutral stimulus is presented to the subject simultaneously with the unconditioned stimulus in one or more opportunities, then an association is created and the NS becomes a conditioned stimulus (CS) that, even in the absence of the US, provokes a conditioned response (CR) like the unconditioned one. The typical example from Pavlov’s original research is the physiological reaction of dogs in the presence of food [46]. A dog naturally salivates (UR) in the presence of food (US). If, for example, a dog is stimulated by the sound of a bell (NS), no reaction in the digestive system is found. However, if the food is accompanied by a bell sound in several opportunities, the dog learns to associate the bell to food. Then, the sound of the bell becomes a CS that provokes salivation in the absence of food (CR).

There are many examples of neuromorphic circuits involving memristors that appear to mimic Pavlovian learning [31,43,44,45,60,61]. Tan et al. [45] note that Pavlovian conditioning comprises three different behaviors: (1) acquisition of the association by training trials where NS and US are either simultaneous or close in time, (2) extinction of the association (forgetting) when CS is applied alone, and (3) recovery by a training process after the last extinction. According to Tan and colleagues, no previous works addressed all three features of classical learning.

**Figure 3:**
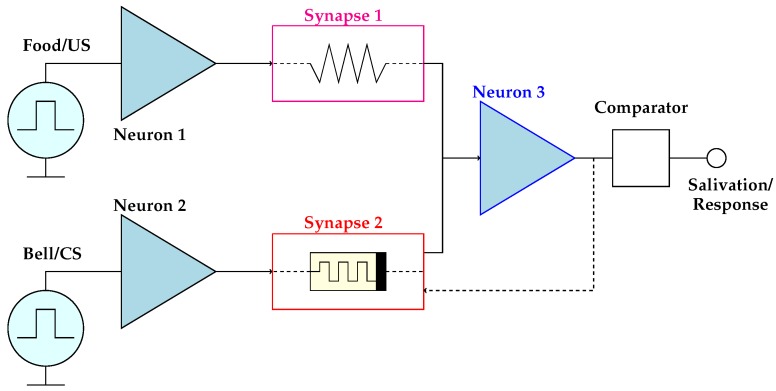
Block diagram of the system used to mimic Pavlovian learning [43].

Figure 3 shows a block diagram of the experimental setup identical to that in Hu et al. [43]. The unconditioned stimulus is fed into neuron 3 through synapse 1. Since the response to the US is innate and assumed to be unchangeable, synapse 1 is simply implemented as a constant resistor. The conditioned stimulus is fed into neuron 3 through synapse 2. Given that actual conditioning occurs in this synapse, its implementation is slightly more complex and it involves a memristor. Moreover, this synapse receives feedback from neuron 3. The output of the experimental setup is a simple comparator that gives a binary signal (salivation/no-salivation) based on the output of neuron 3. In Section 2.3, we show that the simplified model in Equations (2)–(5) is useful to reproduce the essence of classical conditioning when used to emulate the memristor in Figure 3. A detailed description of the experimental setup, including circuits schematics, is given in Section 3.2.

## 2. Results and Discussion

### 2.1. Validation of the Emulation Architecture

**Figure 4:**
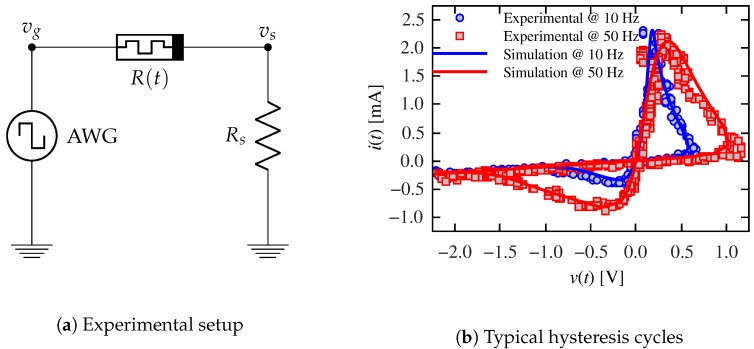
Emulator results: (**a**) experimental setup. The circuit under test is driven by an arbitrary waveform generator (AWG). Current through the memristor is measured as a voltage drop on an in-series resistor $R_{s}=1\;k\Omega$. (**b**) circuit current vs. memristor voltage: simulation (solid line) and measured emulator (points) results. The AWG provides a variable frequency sinusoidal signal with amplitude A=2.5V. The switching thresholds move towards higher voltage values when the input frequency increases. Parameters were set to $\alpha_{\pm }=15\;V^{-1}$, $\delta_{\pm }=0.2\;V$, $R_{\mathrm{on}}=35\;\Omega$, $R_{\mathrm{off}}=9.5\;k\Omega$, $v_{0}=0.3\;V$, and $\tau_{0}=0.01$ s.

We verified the correctness of the emulator design by implementing the memristor model introduced in Section 1.1 and comparing the resulting measurements with numerical simulations. The circuit schematic of the experimental setup and typical measurements are shown in Figure 4. The circuit under test, shown in Figure 4a, is comprised of an arbitrary wave generator that feeds the emulator device with a sinusoidal signal and an in-series measuring resistance that tracks the flowing current. Figure 4b shows experimental results for two driving frequencies. It can be seen that the rate at which the driving signal changes influences the apparent switching threshold [62]. In order to validate the memristor emulator, we solved Equations (2)–(5) numerically. These results are presented in Figure 4b showing a good agreement with the emulator results.

### 2.2. Synapse Mimicking

**Figure 5:**
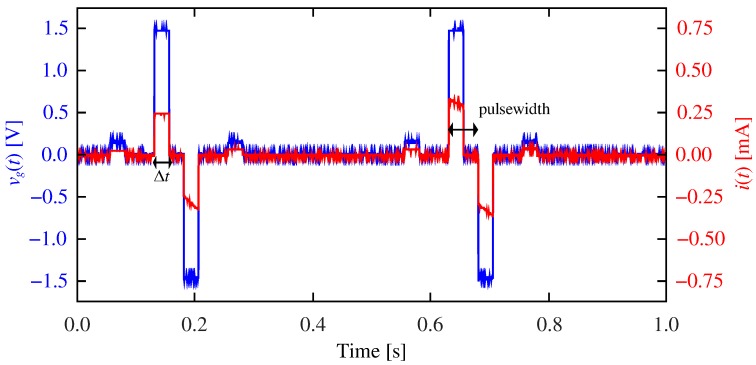
Driving signal $v_{g}$ and current *i* flowing through the emulator. Two 50ms-wide and 1.5V-high stimulus pulses and two 25ms-wide and 200mV-high measurement pulses comprise each period. In this example, Δt=25ms and the pre and postynaptic stimuli overlap for 25ms. Parameters: $\alpha_{\pm }=30\;V^{-1}$, $\delta_{\pm }=0.75\;V$, $R_{\mathrm{on}}=1\;k\Omega$, $R_{\mathrm{off}}=5\;k\Omega$, $v_{0}=0.2\;V$, and $\tau_{0}=10$ s.

Part of the material in this section was already presented in Ref. [50]. The main goal is to reproduce the STDP process by an appropriate pulsing experiment with the memristor playing the role of the synapse. We used a simple circuit comprising an arbitrary waveform generator, a resistor, and the memristor emulator as it is schematized in Figure 4a. We applied a 500ms-period signal consisting of two stimulus pulses, one positive (the presynaptic stimulus) and one negative (the postsynaptic stimulus). The signal also included two measurement pulses, one of them 50ms before the presynaptic pulse and the other 50ms after the postsynaptic stimulus. While the stimuli were 50ms-wide and had an absolute amplitude of 1.5V, the measurement pulse was only 25ms-wide and 200mV high. Pulse duration was partly determined by the frequency limitations of the emulator circuit (see Section 3.1). The measurement pulse amplitude was chosen in order to avoid a significant resistance change. Figure 5 shows a particular example where two stimuli overlap for Δt=25ms. Figure 5 also shows results tracking the current flowing through the emulator. As expected, the transient response of the current corresponds to the resistance change of the emulator. Let us remark that the results in Figure 5, as well as the results in all the remaining figures of this work, were experimentally obtained on the basis of emulated memristors.

**Figure 6:**
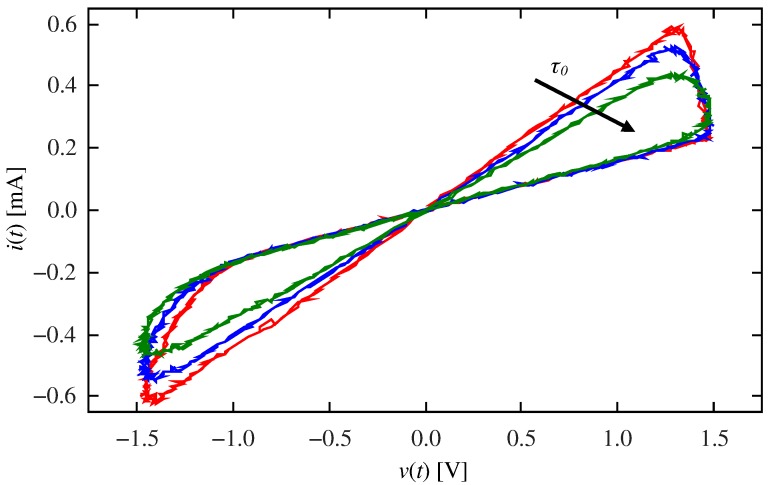
Circuit current vs. memristor voltage: measured emulator results. The AWG provides a 1 Hz sinusoidal signal with amplitude A=1.5V. Parameters were set to $\alpha_{\pm }=30\;V^{-1}$, $\delta_{\pm }=0.75\;V$, $R_{\mathrm{on}}=1\;k\Omega$, $R_{\mathrm{off}}=5\;k\Omega$, and $v_{0}=0.2\;V$. The device’s response time was varied: $\tau_{0}=5,\;10,\;20$ s (red, blue, and green lines, respectively). Extreme memristance values depend on the value $\tau_{0}$.

Having fixed the pulsing protocol, model parameters were chosen on a trial and error basis, aiming to obtain the desired synapse-like behavior: $\alpha_{\pm }=30\;V^{-1}$, $\delta_{\pm }=0.75\;V$, $R_{\mathrm{on}}=1\;k\Omega$, $R_{\mathrm{off}}=5\;k\Omega$, and $v_{0}=0.2\;V$. Since we are interested on the influence of the device’s response time, $\tau_{0}$ was varied. In order to understand the behavior of the memristor with the selected parameters, Figure 6 shows experimental results, measured on the emulator, for different values of $\tau_{0}$. The experimental setup is the same as in Figure 4a, where a sinusoidal signal is applied. The frequency (1 Hz) and amplitude (1.5V) were set to be commensurate to those in the pulsing experiment. It is interesting to compare the resulting curves in Figure 6 with those in Figure 4b. Whereas in the latter case $R_{\mathrm{on}}$ and $R_{\mathrm{off}}$ are attained in each cycle (as evidenced by the same extreme slopes for both driving frequencies), in the former case, the memristance changes between two intermediate values. Moreover, the two extreme resistance values depend on the response time $\tau_{0}$.

**Figure 7:**
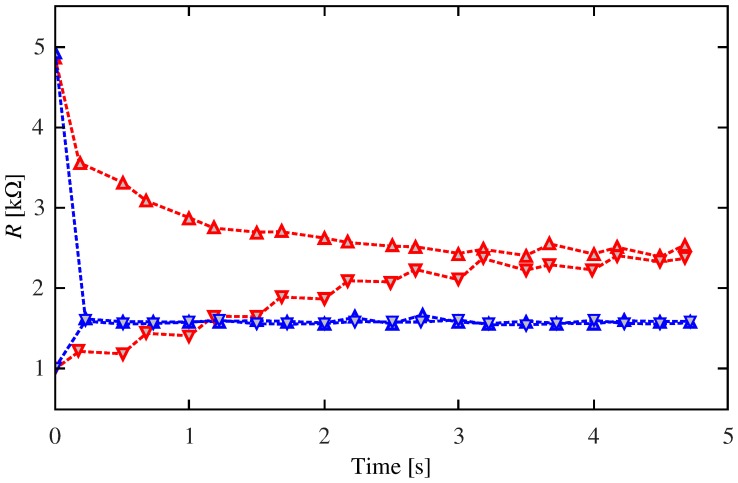
Behavior of the resistance. Initial conditions are indicated by the upside (=$R_{\mathrm{off}}$) and downside (=$R_{\mathrm{on}}$) triangles. Red and blue colors stand for Δt=5ms and Δt=50ms, respectively. All experimental parameters were as in Figure 5 except that $\tau_{0}=5$ s.

**Figure 8:**
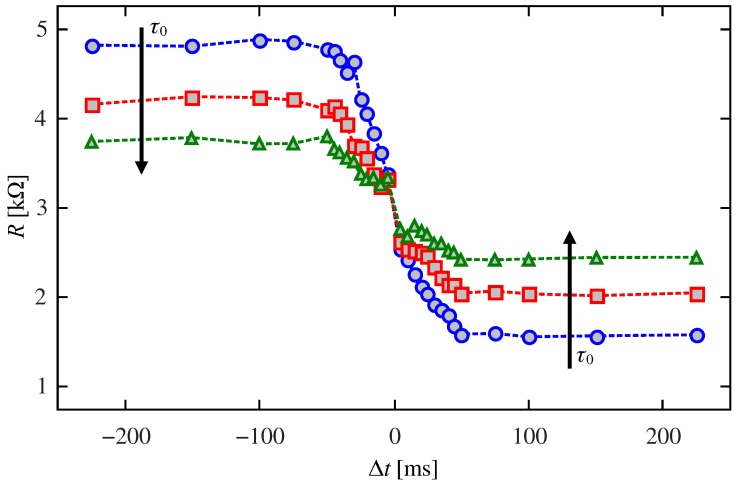
Influence of Δt on the emulator resistance for $\tau_{0}=5$ (blue), 10 (red) and 20s (green). The initial setting was 5kΩ and the remaining experimental parameters were as in Figure 5.

Let us now return to the pulsing protocol in Figure 5. For a fixed Δt, we applied a driving signal that was composed of several periods of the stimulus signal. In this way, we studied the relation between Δt and the resistive change of the device. Figure 7 shows the resistance behavior during the first eight periods for two Δt and a pair of different initial conditions. The figure shows that the final value of the resistance is sensitive to the delay Δt but not to the initial setting. We thoroughly characterized this behavior by exciting the memristor with 20 consecutive periods of the stimulus signal and changing the value of Δt. Figure 8 shows the relation between the final resistance and Δt. In particular, we show results for $\tau_{0}=5$, 10, and 20s (see Equations (2)–(5)). As it can be seen, the behavior depends on whether Δt is smaller or greater than the pulsewidth (50ms). Whenever there is destructive interference between the pre and postsynaptic stimuli (|Δt|<50ms), the final state exhibits a strong dependence on |Δt|. However, no such dependence is observed when |Δt|>50ms and only $\tau_{0}$ influences the final resistance.

**Figure 9:**
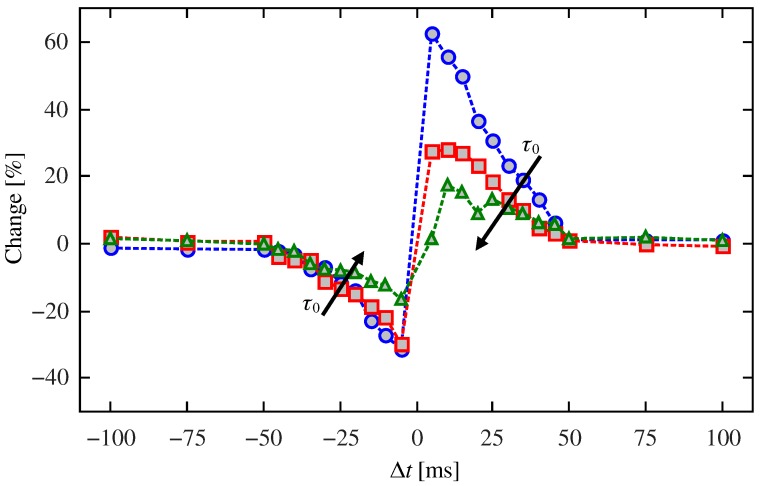
Percentage of change of the resistance vs. Δt for characteristics times $\tau_{0}=5$ (blue), 10 (red), and 20s (green). The larger the $\tau_{0}$, the weaker the learning rule.

Figure 9 shows the influence of Δt and $\tau_{0}$ on the ratio of change of the resistance in relation to its final state. Since the measured behavior of the resistance as a function of Δt is qualitatively similar to that observed in real neurons, we believe that memristive devices that are modeled by this type of dynamic behavior are suitably to be used in neuromorphic circuits inspired by the STDP process. As the final state of resistance is affected by the parameter $\tau_{0}$, it will affect the resistance change ratio. The change of resistance decreases as $\tau_{0}$ increases.

### 2.3. Classical Conditioning

In this section, we show that the simplified model in Equations (2)–(5) is useful to reproduce the essence of classical conditioning. Figure 3 shows a block diagram of the experimental setup identical to that in Ref. [43]. Details of the experimental setup are given in Section 3.2.

**Figure 10:**
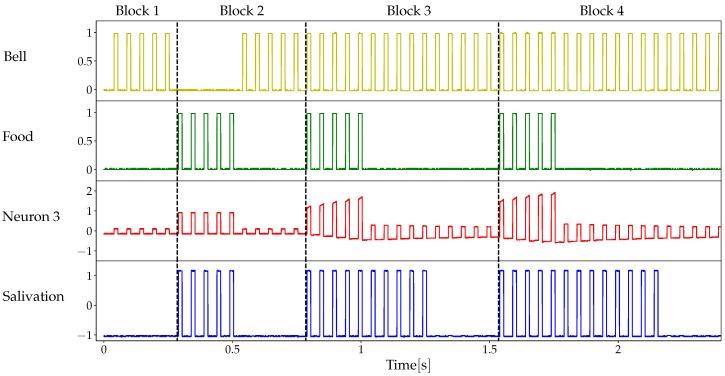
Pavlovian conditioning. When present, stimuli $V_{f}$ (food) and $V_{b}$ (bell) are represented 1 V-high square waves with a 20 Hz frequency and 30% duty cycle. Parameters of the memristor model: $v_{0}=0.2\;V$, $\alpha_{+}=10\;V^{-1}$, $\alpha_{-}=5\;V^{-1}$, $\delta_{+}=0.7\;V$, $\delta_{-}=0.6\;V$.

Figure 10 shows typical results of our experimental setup for Pavlovian conditioning. Results are grouped into four blocks. In the first block, in the absence of association, the bell (signal $V_{b}$) is a neutral stimulus that does not provoke any response (no salivation in the last row). In the second block, the unconditioned stimulus (food, $V_{f}$) is accompanied by the unconditioned response. Although the bell follows immediately after the US has disappeared, no association is produced and there is no response to the neutral stimulus. In the third block, food and bell are simultaneous and the association is acquired: there is a conditioned response to the conditioned stimulus even after the unconditioned stimulus has disappeared. Moreover, after a lapse in which CS is applied alone, the association is forgotten. Finally, in the fourth block, the association is recovered. Note that the forgetting process takes longer than in the third block, which corresponds intuitively to the reinforcement of the association. In summary, all three features described by Tan et al. [45] as necessary for classical conditioning are present, viz. acquisition, extinction and recovery of the association.

**Figure 11:**
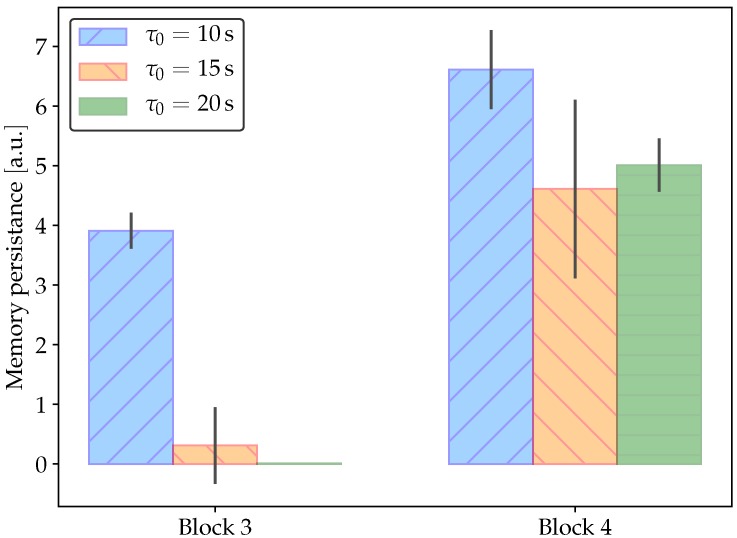
Memory persistence in Pavlovian conditioning. Results correspond to 10 experiments in the same conditions as in Figure 10. As the characteristic response time of the memristor, $\tau_{0}$, increases, it takes longer to produce a strong association between the conditioned stimulus (bell) and the conditioned response (salivation).

One of the advantages of using an emulator instead of an actual memristor is the possibility of changing model parameters easily. It is this advantage that allows us to study the influence of the characteristic response time of the memristor $\tau_{0}$ on learning time and memory persistence. Figure 11 shows results for the same experimental setup as in Figure 10. Memory persistence is measured as the number of input bell pulses, after the food stimulus has disappeared that produces a conditioned response. Since small random variations may produce changes in the measurements, Figure 11 presents results of ten experiments. Although it can be expected that, as $\tau_{0}$ increases, the memory lasts longer, Figure 11 seems to exhibit a different picture. However, the fact is that, as $\tau_{0}$ increases, it takes longer to produce a strong association between the conditioned stimulus (bell) and the conditioned response (salivation). Weaker association for larger memristor response time is reflected in shorter memory persistence. Even in Block 3, no association is learned when $\tau_{0}=20\;s$. Longer memory persistence in Block 4 is due to the strengthening of association after a second round of training.

**Figure 12:**
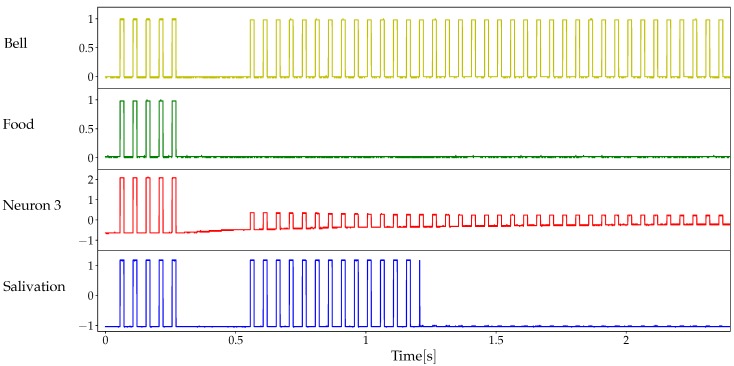
A typical example of the experiments to quantify memory persistence. After both stimuli are interrupted, only the conditioned stimulus (bell’s sound) is re-enabled. In this case, there are five blank spaces (no-stimuli lapse) and the memory persistence is measured as 14. Model parameters are as in Figure 10, except for $\tau_{0}=20\;s$.

**Figure 13:**
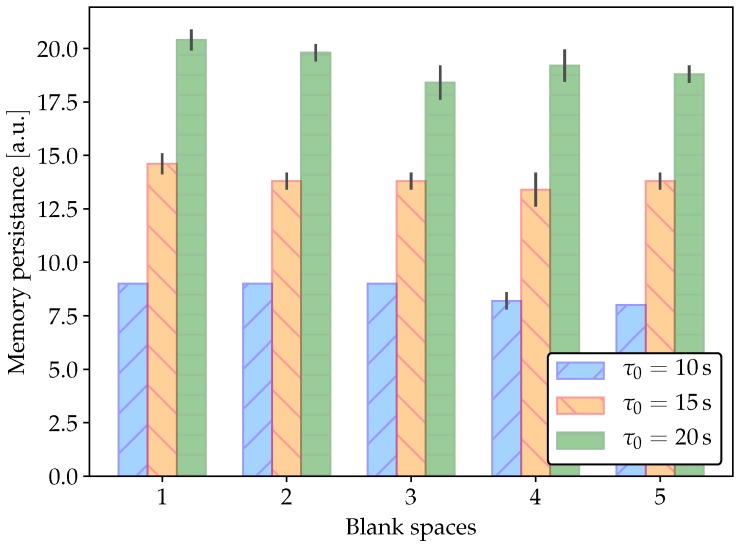
Memory persistence in Pavlovian conditioning. Results correspond to five experiments in the same conditions as in Figure 12.

In order to evaluate memory persistence without the confounding element of learning time, we conducted a different set of experiments where the system departs from a strong association (low memristor resistance, ∼1 kΩ). At the beginning of each experiment, both the unconditioned and conditioned stimuli are present for five pulses. After this association-strengthening period, both stimuli are interrupted for a variable lapse (measured as number of absent stimulus pulses or blank spaces). Finally, only the conditioned stimulus is enabled again after the no-stimuli lapse. Memory persistence is measured as the number of CS pulses that produce a response in this final period of the experiment. Figure 12 shows a typical experiment and Figure 13 shows the results of five experiments. As it can be observed, the learned association persists longer as the characteristic time $\tau_{0}$ increases, as it intuitively expected. Moreover, there is no significant evidence of a stronger forgetting process as the period without stimuli gets longer.

The characteristic response time $\tau_{0}$ varies between the different memristive systems. Amorphous silicon devices present $\tau_{0}$ values of the order of $10^{3}$ to $10^{4}$ s [63,64], HfO${}_{x}$/AlO${}_{x}$ structures of the order of $10^{2}$ s [65], and Ti/HfO/Pt of the order of 1 s [66]. Moreover, many devices present a highly asymmetric behavior between ON/OFF switching times [67]. For these reasons, it is important to perform preliminary characterizations of the neuromorphic circuit to be implemented. Our results suggest that applications, where the information is to be retained for the longest time, should be based on devices with high $\tau_{0}$ value. However, this has the disadvantage that the resulting learning rules are going to be weaker. On the other hand, in applications where the reconfiguration of the connections is dynamic and it is expected to obtain appreciable changes in short times, the design should be based on devices with low $\tau_{0}$ where the learning rules are stronger.

## 3. Materials and Methods

### 3.1. Details of the Emulator Architecture

Figure 2 shows the schematic of the emulator design. A detailed description and analysis of the architecture of the emulator can be found in Ref. [50]. Time steps of the numerical integration algorithm are limited by the computation speed of the microprocessor. For this reason, we resorted to one of the fastest Arduino boards, the Arduino Due with an Atmel SAM3X8E processor running at 84 MHz [68]. The resulting integration time step was ∼400 μs and, hence, frequency of input signals are required to be ≪ 2.5kHz.

We used a Renesas X9C103P [69] potentiometer that accepts bipolar voltage signals and has 100 possible resistance values between ∼35.0Ω and ∼9.5kΩ. Although we found this potentiometer adequate for our current implementation, it would be convenient to upgrade future designs with a higher resolution potentiometer.

The code used to interact with the digital potentiometer was developed by Timo Fager [70]. The X9C103P potentiometer is controlled by an external clock that sequentially changes the resistance by increment or decrement steps. The larger the change in resistance, the longer it takes to realize it due to this sequential programming feature, leading to larger integration time steps. Larger time steps, in turn, limit the highest admissible frequency of the input signals.

Analog-to-digital converters (ADCs) of the Arduino Due admit inputs only between 0.0 and 3.3 V. To prevent damage and malfunction of the microprocessor, there is a signal conditioner circuit to adapt the sensed voltage to adequate signal levels (see Figure 2). Essentially, the signal is buffered, attenuated and biased to comply with the ADC input range.

Measurement errors also limit the emulation accuracy. We found measurement errors much higher than the ADC resolution of the Atmel SAM3X8E microcontroller (12 bits, LSB <1mV) due to several noise sources, suggesting that a better noise-resistant circuit design is needed, especially in signal adaptation stage in Figure 2. One of the possible noise sources is due to digital clock feedthrough. Noise problems were somewhat alleviated with low pass filters.

The model described in Section 1.1 is implemented in Arduino Due using a semi-implicit Euler integration algorithm. Algorithm 1 shows the pseudocode of the main loop. Function setResistance() uses the utilities in Ref. [70]. Function readVoltages() reads the results from the microcontroller’s ADCs and computes the voltage drop on the potentiometer on the basis of the signal adaptation circuit (see Figure 2). The remaining functions are explained in Algorithms 2 and 3.

**Algorithm 1** Model implementation in Arduino Due: Main loop
**while** True **do**      Δt = timeMeasure()                              ▹ Computes the actual integration time step    *v* = readVoltages()                             ▹ Reads voltage adapted at the ADC’s input    *R* = updateResistance(*v*,Δt)      ▹ Integrates the differential equation of the model    setResistance(*R*)                                                 ▹ Sets the potentiometer resistance
**end while**



**Algorithm 2** Model implementation in Arduino Due: Integration time step
**function**timeMeasure      *t* = micros() ▹ Read microcontroller’s running time in microseconds    $\Delta t=t-t_{\mathrm{old}}\;$▹ Time step    $t_{\mathrm{old}}=t$          **return**Δt  
**end function**



**Algorithm 3** Model implementation in Arduino Due: Numerical Integration                        
**function**updateResistance(*v*,Δt)      $\Gamma^{+}=\frac{1}{1+e^{-\alpha_{+}\left(v-\delta_{+}\right)}}$        $\Gamma^{-}=\frac{1}{1+e^{-\alpha_{-}\left(v+\delta_{-}\right)}}$        $\lambda =\min\left\{\Gamma^{-},\max\left[\lambda_{\mathrm{old}},\Gamma^{+}\right]\right\}$        $\tau =\tau_{0}\exp\left(-\frac{\left|v\right|}{v_{0}}\right)$        $w=\frac{\tau w_{\mathrm{old}}+\Delta t\lambda }{\Delta t+\tau }$        $R=R_{\mathrm{on}}w+R_{\mathrm{off}}\left(1-w\right)$        $w_{\mathrm{old}}=w$        $\lambda_{\mathrm{old}}=\lambda$          **return***R*  
**end function**



### 3.2. Details of the Conditioned Learning Experiment

**Figure 14:**
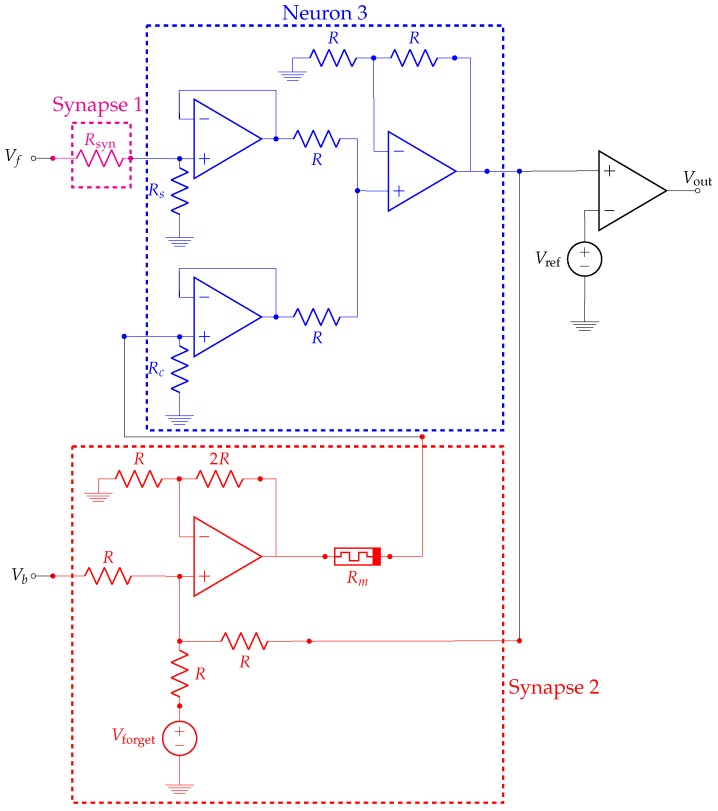
Circuit schematic of the system used to mimic Pavlovian learning. Only the output of the input neurons is represented: $V_{f}$ is the response of neuron 1 to the unconditioned stimulus (i.e., food) and $V_{b}$ is the response of neuron 2 to the conditioned stimulus (i.e., bell’s sound)—cf. Figure 3. Resistance values: R=3.3kΩ, $R_{\mathrm{syn}}=220\;\Omega$, $R_{s}=2.2\;k\Omega$ and $R_{c}=2\;k\Omega$. Constant voltages: $V_{\mathrm{forget}}=600\;mV$, $V_{\mathrm{ref}}=232\;mV$.

Figure 3 shows a block diagram of the experimental setup identical to that in Ref. [43] and Figure 14 shows a schematic of our implementation. We must note that Figure 14 shows only the outputs of neurons 1 and 2 in Figure 3: $V_{f}$ is the response of neuron 1 to the unconditioned stimulus (i.e., food) and $V_{b}$ is the response of neuron 2 to the conditioned stimulus (i.e., bell sound).

The unconditioned stimulus $V_{f}$ is fed into neuron 3 through synapse 1. Since the response to the US is innate and assumed to be unchangeable, synapse 1 is simply implemented as a constant resistor $R_{\mathrm{syn}}$. The conditioned stimulus $V_{b}$ is fed into neuron 3 through synapse 2. Since actual conditioning occurs in this synapse, its implementation is slightly more complex and it involves an emulated memristor $R_{m}$ instead of a constant resistor. Since the strength of the input to neuron 3 depends on the voltage divider formed by $R_{c}$ and $R_{m}$, the input becomes stronger as $R_{m}$ decreases. The constant voltage source $V_{\mathrm{forget}}$ tries to force the memristor in high resistance values. In this sense, $V_{\mathrm{forget}}$ acts as a forgetting drive that is always present. The state of $R_{m}$ can also be altered by the feedback from the output of neuron 3, which is the actual source of association between salivation and the CS.

Simple calculations show that the voltage drop on the memristor is

(6) $$V_{m}=\frac{R_{s}}{R_{s}+R_{\mathrm{syn}}}V_{f}+V_{b}+V_{\mathrm{forget}}.$$

Observe that $V_{m}$ is independent of $R_{m}$ and it depends only on the stimuli. This fact implies that the learning (or forgetting) process is independent of the state of the association between NS/CS and the response.

The output of neuron 3 is fed into a comparator in order to obtain a binary output ($V_{\mathrm{out}}$) such that a high level corresponds to a (conditioned or unconditioned) response to stimuli.

## 4. Conclusions

It has been argued in the literature that diffusive memristor devices may mimic the behavior of synapses. In this work, we presented a computationally-efficient simplification of an accurate and compact model of such devices. We believe that this model can be very useful in the study of complex neuromorphic circuits and we present its application to two simple examples.

The proposed model was used in a memristor emulator composed of a digital potentiometer and a microprocessor. The main advantage of emulation over simulation is its ability to interact with real-world circuits. In order to validate the correct operation of the emulator, several numerical simulations of a very simple circuit were made under different conditions, finding a good agreement with the experimental results. Although the implemented emulation architecture is simple, it has some limitations. In particular, it is based on a microprocessor with relatively low computing capacity. Future work with more complex circuits or a larger number of emulated memristors will require a faster microprocessor.

The emulated memristor was shown to mimic the Spike-Timing-Dependent Plasticity behavior of synapses. Moreover, it was found that the response time parameter of the memristor, $\tau_{0}$, affects the resistance change ratio in the STDP process. The larger $\tau_{0}$, the lower the change of resistance.

Finally, we introduced a memristor-based neuromorphic circuit that exhibited the main characteristics of Pavlovian conditioned learning. We also explored the influence of the response time $\tau_{0}$ in learning and memory persistence. In general, the larger $\tau_{0}$, the longer it takes the system to learn. However, once the conditioned response has been learned, a larger $\tau_{0}$ leads to a longer memory persistence.
